# CD47-targeting antibodies as a novel therapeutic strategy in hematologic malignancies

**DOI:** 10.1016/j.lrr.2021.100268

**Published:** 2021-09-14

**Authors:** Jennifer Sun, Yixuan Chen, Berit Lubben, Ola Adebayo, Barbara Muz, Abdel Kareem Azab

**Affiliations:** a Department of Radiation Oncology, Cancer Biology Division, Washington University in St. Louis School of Medicine, 4511 Forest Park Ave, St. Louis, MO 63108, USA; b Department of Biomedical Engineering, Washington University in St. Louis McKelvy School of Engineering, St. Louis, MO, USA,

**Keywords:** Immunotherapy, CD47, Checkpoint inhibition, Macrophage, Hematologic malignancies

## Abstract

CD47 is a surface glycoprotein expressed by host cells to impede phagocytosis upon binding to macrophage SIRPα, thereby represents an immune checkpoint known as the “don't-eat-me” signal. However, accumulating evidence shows that solid and hematologic tumor cells overexpress CD47 to escape immune surveillance. Thus, targeting the CD47-SIRPa axis by limiting the activity of this checkpoint has emerged as a key area of research. In this review, we will provide an update on the landscape of CD47-targeting antibodies for hematological malignancies, including monoclonal and bi-specific antibodies, with a special emphasis on agents in clinical trials and novel approaches to overcome toxicity.

## Abbreviations

V_H_: heavy chain variable region
V_L_: light chain variable region
Fab: fragment antigen-binding
Fv: variable fragment
Fc: fragment crystallizable region
scFv: single chain variable fragment.

## 1 Introduction

The tumor microenvironment (TME) contains cellular and non-cellular components such as immune cells, blood vessels, extracellular matrix, cytokines, growth factors, etc. that play critical roles in the development and progression of cancer [1], [2], [3]. The TME confers a hostile environment where immune responses are suppressed and exhausted, mediated by suppressive cell types including regulatory T cells, tumor-associated macrophages (TAMs), and myeloid-derived suppressor cells [4], [5], [6], [7]. Therefore, increasing attention has been focused on elucidating the interplay between TME immune cells and cancer cells, and discovering targetable interactions for therapy [8, 9]. Cancer immunotherapy is a popular class of therapy that focuses on the repair, stimulation, or enhancement of the body's natural immune responses to fight cancer. The recovery of immune surveillance by immunotherapy has the potential for durable response, which can serve as a powerful tool in combination with chemotherapy and other novel TME-targeting approaches [10].

### 1.1 Checkpoint immunotherapy

Among the most promising approaches for cancer immunotherapy is immune checkpoint blockade. Immune checkpoints are inhibitory pathways that help keep immune responses “in check” and prevent immune cells from killing normal cells, such as the “don't kill me” signal in T cells [11]. However, cancer cells were found to overexpress immune checkpoint proteins on their surface [12], making them less visible to immune surveillance [13, 14]. Blocking these checkpoints on cancer cells effectively releases the “brakes” on the immune system, allowing for a restored anti-tumor immune response [15]. Examples of checkpoints that negatively regulate T-cell immune functions include programmed cell death protein 1 (PD-1; on T cells) and its ligand (PD-L1; on target cells), as well as cytotoxic T-lymphocyte-associated antigen 4 (CTLA-4; on T cells) and its ligands (B7–1/B7–2; on target cells) [16]. Immune checkpoint blockade using monoclonal antibodies (mAbs) as inhibitors against these targets has become a paradigm-shifting treatment in solid tumors and blood cancers, enabling patients to produce an effective anti-tumor response [17].

### 1.2 Macrophages in cancer

While T-cell based immunotherapy has gained the lead, the lack of T cell infiltration, T cell activation, and expression of tumor antigen lead to variable and suboptimal response, which warrants the development of therapies that transform the immunosuppressive “cold” TME [18]. Macrophages are key players in the innate immune system. As “professional eaters” of the immune system, they serve as the first-line of defense, specializing in the rapid detection, phagocytosis, and destruction of foreign substances, microbes, cancer cells, and other harmful organisms [19, 20]. Macrophages also function as antigen presenting cells, which induce and direct adaptive immune response (such as in T cells and B cells) [21]. Additionally, macrophage population can rapidly expand by recruitment of monocytes to inflammation and tumor sites [9].

TAMs are a prominent immune population within the TME. Rather than contributing to the immune response against tumor cells, TAMs are often found to exhibit pro-tumor properties including supporting chemoresistance, tumor proliferation and survival, angiogenesis, immunosuppression, and metastasis [22], [23], [24]. Targeting TAMs represents a novel strategy for cancer immunotherapy, which has the potential to indirectly stimulate cytotoxic T cell activation and recruitment, and synergizes with checkpoint inhibitors and chemotherapies [25, 26].

### 1.3 CD47-SIRPa checkpoint

A major macrophage immune checkpoint is the CD47-SIRPa checkpoint. CD47 is a transmembrane protein expressed across a wide range of normal cell types, and it functions mainly as a marker for macrophages to differentiate “self” from “non-self” [27, 28]. The signal regulatory protein α (SIRPα) is regularly expressed on myeloid cells [29]. Binding of CD47 to SIRPα receptor on the surface of macrophages leads to downstream signaling within the macrophages, resulting in inhibition of phagocytic activity. Thus, the CD47-SIRPa interaction is also known as the “don't-eat-me” signal.

Accumulating evidence shows that various solid and hematologic malignancies overexpress the CD47 protein on the surface as a protective “self-marker” [30]. Thus, targeting the CD47-SIRPa axis by limiting the expression of the “don't-eat-me” signal has emerged as a key area of research. Currently, there are a wide range of studies aiming to inhibit the checkpoint using various strategies, including anti-CD47 antibodies, anti-SIRPα antibodies, and soluble SIRPα proteins [31, 32]. The best characterized therapies targeting this checkpoint are anti-CD47 antibodies, which have proven effective in inducing phagocytosis of tumor cells in vitro as well as inhibiting growth of both hematologic and solid tumors [33], [34], [35]. Additionally, there are various Phase 1 and 2 clinical trials investigating the therapeutic efficacy of anti-CD47 antibodies on hematologic and solid malignancies as single agent or combination treatment [36, 37].

### 1.4 CD47 in hematological malignancies

Hematological malignancies comprise three major categories: leukemia, lymphoma, and myeloma. Increasing body of evidence indicates the significance of CD47 in pathogenesis and progression of various hematological malignancies, validating CD47 as a candidate for targeted therapy [35, 38, 39].

In an array of non-Hodgkin lymphoma (NHL) subsets, CD47 was found to be increased on primary NHL cells compared to B cells, which was an independent predictor for worse clinical outcomes [33]. In cutaneous T cell lymphoma (CTCL), TME with higher CD47 checkpoint inhibition correlated with advanced disease state [40].

In acute myeloid leukemia (AML), it was reported that the self-renewing leukemia stem cells more highly expressed CD47 than bone marrow hematopoietic stem cells (HSCs) and multipotent progenitor (MPP) cells. Additionally, high CD47 expression at time of diagnosis associated with inferior survival outcomes [41]. In myelodysplastic syndrome (MDS), CD47 expression is high in high-risk patients compared to low-risk MDS and controls, indicating CD47 as a negative clinical prognosis marker [42]. Human acute lymphoblastic leukemia (ALL) patient samples showed 2-fold higher CD47 expression compared to normal bone marrow [43], and higher CD47 level independently correlated with worse overall survival [44].

In multiple myeloma (MM), transition from the precursor disease monoclonal gammopathy of undetermined significance (MGUS) to MM is associated with a significant increase in the population of plasma cells expressing CD47 [35, 45]. One study found that 73% of MM patients had overexpression of CD47 compared to non-myeloma cells [46]. Additionally, CD47 mRNA expression directly correlated with disease progression and primary MM cells had an 8-fold higher surface CD47 expression compared to other bone marrow populations [35].

**Fig. 1:**
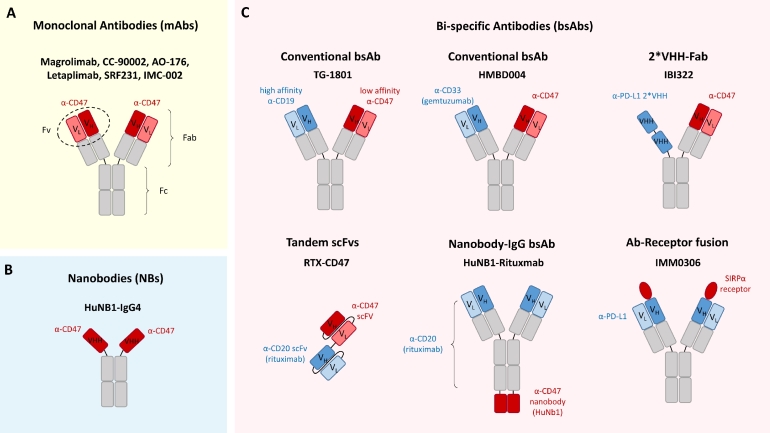
**Structures of anti-CD47 antibody therapeutic agents in preclinical and clinical development. (A)** Monoclonal antibodies (mAbs): conventional IgG mAbs including Magrolimab, CC-90002, AO-176, IBI188, SRF231, and IMC-002. **(B)** Nanobodies (NBs) such as HuNB1-IgG4. **(C)** Bi-specific antibodies (bsAbs) including TG-1801, HMBD004, IBI322, RTX-CD47, HuNB1-Rituximab, and IMM0306.

In this review, we will provide a comprehensive update on CD47-targeting antibodies for hematological malignancies, including monoclonal and bi-specific antibodies, with a special emphasis on agents in clinical investigation (Fig. 1). Finally, we will discuss future perspectives regarding of CD47-targeted therapy, including the issue of off-target toxicities in patients, as well as the promising potential for combination therapy.

## 2 CD47 mAbs

### 2.1 Magrolimab/Hu5F9-G4

Magrolimab, previously known as Hu5F9-G4, is a humanized IgG4 anti-CD47 mAb that is in various stages of clinical trials for hematologic malignancies as well as solid tumors [47]. Magrolimab tightly binds to human CD47 antigen and induces phagocytosis of tumor cells in a non-antibody dependent cellular cytotoxicity (ADCC) mechanism [48].

Blockade of CD47 using Magrolimab demonstrated eradication of leukemic cells in AML xenograft model in vivo, resulting in prolonged survival [49]. In a Phase 1 dose-escalation study in relapsed/refractory (R/R) AML patients (NCT02678338), the side effect of Magrolimab was evaluated. Although patients were asymptomatic from the adverse effect, all patients experienced red blood cell (RBC) agglutination and anemia, due to phagocytosis of CD47 expressing RBS. 95% of patients were transfused since the treatment started. However, no severe side effects, such as hemolysis, were observed [50].

In a Phase 1 trial evaluating efficacy of Magrolimab in R/R AML and MDS patients (NCT03248479), Magrolimab monotherapy did not achieve a robust response despite the preclinical success [51, 52]. However, administering Magrolimab in combination with azacitidine (AZA), a nucleotide analog and hypomethylating agent (HMA), showed a better result, achieving 53% complete remission (CR) overall, while 10% of patients achieved morphological leukemia free state (MLFS) with Magrolimab alone [51]. Magrolimab appeared to shorten treatment response time to AZA, while the safety profile was similar to AZA alone [53, 54]. The combination treatment induced a robust and durable response in both untreated MDS and AML patients [53]. In MDS patients, the objective response rate (ORR) to AZA + Magrolimab was 91%, and responding patients maintained for at least six months [54]. In an expanded cohort of untreated AML patients, ORR to the combination treatment was 65% overall and 71% for the *TP53* mutant subgroup who were high-risk and refractory-prone [54].These encouraging results in early clinical trials have laid the groundwork for further clinical investigations. The efficacy of Magrolimab monotherapy or in combination with AZA will be further examined in an expanded cohort of untreated MDS patients in a randomized Phase 3 ENHANCE study (NCT04313881).

The synergistic effect of Magrolimab in combination with other therapies was further examined in multiple clinical trials. Combination of Venetoclax (VEN), a BCL-2 inhibitor, and decitabine/AZA led to encouraging results in untreated AML patients, in which the ORR of 400-mg VEN + AZA treatment group was 76% [55]. Adding Magrolimab to VEN + AZA combo is currently investigated in a Phase 1b/2 clinical trial to treat untreated, recurring, and refractory AML patients (NCT04435691). Moreover, a Phase 2 multi-arm study will evaluate different combination regimens more comprehensively (NCT04778410). It aims to test the combination of Magrolimab with VEN + AZA and two new combinations of Magrolimab with Mitoxantrone + Etoposide + Cytarabine (MEC, a chemotherapy regime) or with CC-486 (oral AZA) in previously untreated AML and in R/R AML. Finally, a Phase 1 study to evaluate the combination of Magrolimab with Atezolizumab, an anti-PD-L1 agent, in R/R AML patients was recently completed, but the results are not yet available (NCT03922477).

Additionally, Magrolimab has demonstrated potential therapeutic efficacy in NHLs. A preclinical study showed that Magrolimab re-sensitized large cell lymphoma cell line resistant to rituximab, increasing phagocytosis by 80% compared to rituximab alone [56]. A Phase 1 clinical trial (NCT02953509) corroborates the efficacy of the combination of Magrolimab with Rituximab in R/R NHL patients [56]. In a small cohort of R/R Diffused Large B-Cell Lymphoma (DLBCL) and follicular lymphoma (FL) patients, ORR reached 50% among all, 71% in FL patients, and 40% in DLBCL patients. In addition, the regime produced durable response – the median duration of response has not yet reached after more than 6- and 8-months follow-ups for DLBCL and FL, respectively. The result is especially significant for DLBCL, which is the most common subtype of NHL but lacks effective salvage therapy options [57], where the response to salvage therapy was minimal (ORR of 26%, median OS of 6.3 months) [58]. Similarly, there is no clear recommendation on therapeutic options for rituximab-refractory patients [59]. Additionally, a multi-arm Phase 1 PRISM study investigating different combination strategies in R/R DLBCL patients was recently completed, but the results were not yet available (NCT03527147).

Aside from B-cell lymphomas, a randomized Phase 1b/2 study is testing the combination of Magrolimab with Mogamulizumab, an anti-CCR4 agent, compared to Mogamulizumab alone, in R/R T-cell lymphoma (NCT04541017).

### 2.2 CC-90002

CC-90002 is a humanized IgG4 anti-CD47 mAb. Preclinically, CC-90002 showed efficacy across a plethora of solid and hematologic malignancies [60]. Specifically, CC-90002 blocked CD47-SIRPα interaction with high affinity, enabling macrophage-mediated killing of AML, ALL, MM cell lines and primary AML patient samples. Anti-tumor activity was demonstrated in MM xenograft models, in which binding of CC-90002 to tumor cells as well as recruitment of M1-polarized F4/80 macrophages contributed to tumor regression [60, 61]. Rapid and substantial tumor reduction was also seen in AML xenograft models [62].

A Phase 1 clinical trial investigated safety, tolerability, and efficacy of CC-90002 as monotherapy was carried out in R/R AML and high-risk MDS patients (NCT02641002). However, this study was terminated due to the lack of preliminary monotherapy activity and discouraging profile for dose escalation [62].

Additionally, a Phase 1 clinical trial tested the combination CC-90002 with Rituximab in CD20-positive R/R NHL (NCT02367196) [63]. Early results showed that the combination treatment resulted in an overall response rate of 13% and disease control rate of 25%, compared to lack of response in CC-90002 alone. While clinical efficacy was limited, the combination demonstrated tolerability; dose-limiting thrombocytopenia was common but hemolysis was not observed [63]. Final results from the study are yet to be published.

### 2.3 AO-176

AO-176 is a humanized IgG2 anti-CD47 mAb with attractive biologic characteristics, including preferential binding to tumor cells, minimal binding to RBCs, and a non-antibody dependent cellular cytotoxicity (ADCC) based killing mechanism [64], therefore, it has the potential to overcome toxicities seen in previous CD47-targeting agents.

Pre-clinically, in vitro, AO-176 was shown to bind to T-ALL and B lymphoma cell lines with higher affinity than isotype control. Importantly, minimal binding to healthy donor RBCs were detected. The antibody also showed preferential binding to tumor cells compared with platelets, T cells, RBCs, and endothelial cells. Further, the induction of cell death was selective for tumor cells. The in vivo antitumor efficacy was studied in lymphoma xenograft, which resulted in 25% tumor inhibition at lowest dose of 1 mg/kg compared to isotype control, and achieved 73% and 82% inhibition at 10 and 25 mg/kg, respectively. Additionally, tolerability and hematologic changes were determined in cynomolgus monkeys, which presented reduced effect of RBC parameters, similar to in vitro results. AO-176 was well tolerated and no adverse side effects were observed, thus representing a valuable antibody candidate which could achieve favorable pharmacokinetics (PK) and safety profiles in humans.

AO-176′s anti-tumor activity was also evaluated in MM [65]. As a single agent, AO-176 resulted in durable inhibition of tumor growth in MM xenograft mice models. Additionally, combination treatment with other anti-MM therapies including bortezomib, daratumumab, lenalidomide, or pomalidomide demonstrated further extended survival compared to AO-176 alone. These encouraging pre-clinical results have culminated into a currently recruiting Phase 1/2 clinical study evaluating AO-176 as monotherapy and as combination with bortezomib/dexamethasone in MM patients (NCT04445701).

Related to AO-176, Vx1000R is a mouse anti-human CD47 antibody [37]. It was tested as a therapeutic antibody for MM. In 3D tissue engineered bone marrow (3DTEBM), the inhibition of CD47 by Vx1000R induced a 75% killing of MM cells compared to no treatment and isotype controls. The effect began as early as 4 h, much faster than T cell checkpoint-inhibition-mediated killing. Durable effect was observed over 24 h. However, without the presence of macrophages, neither the IgG control nor Vx1000R induced cytotoxicity in MM cells. Moreover, neither IgG nor Vx1000R killed MM cells with the presence of macrophages in 2D cultures. The difference in phagocytosis and killing of MM between 2D and 3D cultures may be due to the 3DTEBM's patient-derived matrixed structure, which better simulates the complex TME conditions in vivo and drug responses observed in patients [35, 66].

### 2.4 Letaplimab

Letaplimab, also known as IBI188, is a fully human IgG4 anti-CD47 mAb. In vitro, Letaplimab binds to cancer cell lines with comparable affinity with magrolimab, and enhanced macrophage phagocytosis in a dose-dependent manner [67]. In both NHL and AML xenograft models, Letaplimab monotherapy showed considerable response. The combination of Letaplimab with AZA showed improved efficacy compared to AZA alone, possibly through increased CD47 expression by AZA [67].

Multiple clinical trials are currently underway evaluating Letaplimab as monotherapy or combination therapy in an array of hematologic malignancies. A Phase 1 clinical trial aims to evaluate the safety, tolerability, and efficacy of Letaplimab as monotherapy in lymphoma (NCT03763149). Preliminary data showed well tolerance at 1 mg/kg priming dose with maintenance dose up to 30 mg/kg. Letaplimab overcame sink at 10 mg/kg and higher, with T cell and RBC receptor occupancy around 90% after multiple administrations, with preliminary evidence for durable anti-tumor response in some patients [68]. Other Phase I trials include Letaplimab in combination with rituximab in advanced lymphoma (NCT03717103), with AZA in AML (NCT04485052), and with AZA in newly diagnosed higher risk MDS (NCT04485065).

### 2.5 SRF231

SRF231 is a fully human IgG4 anti-CD47 mab. The defining feature of SRF231 in pre-clinical models was its ability to bind to CD47 with high affinity without inducing hemagglutination or RBC phagocytosis. SRF231 promotes macrophage-mediated phagocytosis of several hematologic primary tumor samples and cell lines in vitro [69]. In vivo efficacy was evaluated using preclinical murine xenograft models of hematologic malignancies, which led to profound tumor growth inhibition in MM, DLBCL, and Burkitt's lymphoma as a single agent and combinations with opsonizing antibodies. Notably, in the Raji xenograft model, a single-agent therapy led to abrogation of tumor growth [69]. Another in vivo study showed increased macrophage infiltration, induction of macrophage cytokines, and induction of phagocytosis through CD47. SRF231’s activity was also found to be dependent on binding to the macrophage activating FcγR CD32a through its Fc domain [70].

SRF231 is currently under clinical investigation as monotherapy in advanced solid tumors and hematologic cancers (NCT03512340). Preliminary data reported that patients with solid tumors show more than 90% occupancy throughout the dosing period, and that SRF231 can be safely administered although there was no complete or partial responder [71]. The final results are yet to be published.

### 2.6 IMC-002

IMC-002 is a fully human IgG4 anti-CD47 mAb. IMC-002 showed optimal affinity to CD47 ligand in multiple types of CD47-expressing cancer cell lines in a preclinical study [72]. IMC-002 also showed selective binding to CD47 on cancer cells but not on RBCs, avoiding agglutination in vitro [50]. Such encouraging result led to a first-in-human Phase 1 clinical trial for IMC-002 in metastatic/locally advanced solid tumors and R/R lymphoma patients (NCT04306224).

### 2.7 Promising CD47 mAbs in preclinical stages

CD47-B is a fully human IgG1 anti-CD47 mAb which have increased phagocytosis of CD47-expressing cells by human macrophages and demonstrated anti-tumor activity in leukemia mouse models. CD47-B does not have significant hemagglutination activity and low platelet-aggregation activity [73]. This feature may indicate an improved safety profile in future clinical trials.

AMMS4-G4 is a fully human IgG4 anti-CD47 mAb. It is developed based on ZF1, a fully human anti-CD47 antibody isolated via a phage display library screening [74]. It demonstrated a robust affinity to recombinant CD47 (KD = 1.19 nM) and induced phagocytosis of AML and ALL cells by human macrophages in vitro. Compared with magrolimab in ALL and AML mice models, AMMS4-G4 demonstrated similar efficacy, extending the life span of mice significantly. Interestingly, while magrolimab induced hemagglutination on cynomolgus monkey RBCs and human RBCs in vitro, AMMS4-G4 did not, only causing reversible anemia when tested in cynomolgus monkeys in vivo [75]. Similar to CD47-B, AMMS4-G4 has reduced hemagglutination and possibly an improved safety profile in future clinical trials [75].

4D10 is a chimeric antibody with variable regions grafted onto human IgG4 [76]. The amino acid sequence presented 64% homology and 54% homology in the VL region compared to magrolimab and CC-90,002 respectively; there was a 69% and 57% homology in the VH region. In preclinical study, it displayed potent macrophage-mediated phagocytosis in AML xenograft model. Notably, no T-cell death or hemagglutination was seen in vitro and only limited hematologic toxicity in vivo in hCD47/hSIRPα double knock-in model.

## 3 CD47 bi-specific antibodies (bsAbs)

CD47 is heavily expressed on non-tumorous body cells such as erythrocytes and platelets [77]. Cross-linking CD47 on different erythrocytes by mAbs leads to hemagglutination and serious side effects [50, 63]. One strategy to overcome this hurdle is the simultaneous targeting of another tumor antigen, which increases the specificity of treatment to tumor cells and limit interaction with other CD47 expressing normal cells [78]. BsAbs work by combining the binding sites of multiple targets into one antibody [79]. There are several CD47-targeted bsAbs under preclinical development, with a few commencing in clinical trials [80].

### 3.1 TG-1801 (CD47xCD19)

TG-1801 is a fully humanized IgG1 anti-CD47/CD19 bsAb in the κλ body format. It consists of a single heavy chain, along κ and λ light chains that each recognize CD47 or CD19, which self-assembles into a functional antibody [78]. The high affinity CD19-binding arm anchors onto B cells and the low affinity anti-CD47 arm can then co-bind cells. Importantly, the production process of such κλ body does not require extensive engineering as traditional bsAbs. In addition to inducing phagocytosis by inhibition of CD47 checkpoint, the intact Fc region of TG-1801 allows further macrophage recruitment and activation. Studies in non-human primates have shown that TG-1801 to have a good PK profile without causing hemotoxicity [81]. Additionally, in a preclinical study in B-cell lymphoma, TG-1801 showed synergy with B-cell targeted mechanisms such as umbralisib (TGR-1202; PI3Kδ and CK1ε inhibitor) and ublituximab (TG-1101; a chimeric anti-CD20 mAb) in vivo, which warrants for further evaluation. [82].

A Phase 1 clinical trial involving the use of TG-1801 is currently undergoing for patients with B-cell lymphomas (NCT03804996). The clinical trial includes a combination treatment of TG-1801 with ublituximab. Additionally, a Phase 1b trial is underway to study TG-1801 as monotherapy or in combination with ublituximab in B-cell lymphomas and CLL (NCT04806035). No clinical data has been reported yet.

### 3.2 IMM0306 (CD47xCD20)

IMM0306 is an anti-CD47/CD20 bsAb of the antibody-receptor fusion format, which combines an anti-CD20 mAb with the extracellular domain of SIRPα receptor [83]. Precinical study shows strong binding to both CD20 and CD47 targets, and elicits a stronger ADCC compared to rituximab alone. Moreover, IMM0306 has no binding activity toward human RBCs and significantly inhibited tumor growth. Toxicity study in non-human primates demonstrated encouraging PK profile with minimal hemotoxicity after multiple administrations [84]. Preclinical efficacy and evidence for RBC avoidance has culminated in a Phase I clinical trial, which will study the safety and PK of IMM0306 as monotherapy in patients with R/R CD20-positive B-cell NHL (NCT04746131).

### 3.3 RTX-CD47 (CD47xCD20)

RTX-CD47 is a bi-specific tandem scFv comprises of an anti-CD20 scFv from rituximab, linked to an anti-CD47-blocking scFv [85]. Treatment with RTX-CD47 selectively triggered phagocytosis of CD20 + /CD47 + double positive cells, but not CD47 + cells. The phagocytosis effect did not require an FcR signaling. In a phagocytosis assay, RTX-CD47 induced macrophage-mediated phagocytosis of CD20 + *B* lymphoma cell lines. Additionally, treatment also was able to induce phagocytosis of primary malignant B cell phagocytosis by autologous macrophages. Importantly, this effect is CD20-restricted, since co-inhibition with excess amount of RTX-antibody fragments inhibited phagocytosis [85]. While the small size of RTX-CD47 may be challenged with poor PK [86], the dual targeting strategy in a tandem scFv format allowed select targeting of cancer cells and avoided excessive activation and ADCC due to absence of Fc region. More pre-clinical investigations are warranted to study the tumoricical ability of RTX-CD47.

### 3.4 HuNb1-Rituximab (CD47xCD20)

Nanobodies (NBs) are single-domain antibody fragments derived from camelid heavy-chain antibodies, which are advantaged with small size (12 kDa), high affinity, stability, and ease to modify [87]. NBs represent a novel form of therapeutic agent and helps mitigate the problems posed by mAbs.

HuNb1 is high affinity NB that is specific for human CD47 and exhibits low binding to human RBCs. HuNB1-IgG4 is a humanized version of NB1-IgG4 which is an anti-CD47 NB fusion protein [87]. In a lymphoma mouse model, treatment with HuNB1-IgG4 alone induced significant tumor apoptosis and necrosis in a dose-dependent manner. In terms of toxicities, no significant RBC hemagglutination at HuNB1-IgG4 concentrations ranging from 0.98 to 4000 nM. Cynomolgus monkeys treated with a low priming dose followed by a high treatment dose displayed no significant adverse effect in vivo [87].

To further enhance the therapeutic properties of HuNB1, a bsAb was constructed containing HuNB1 and rituximab. This anti-CD47/CD20 bsAb demonstrated preferential binding to Raji cells versus erythrocytes and a more potent anti-lymphoma activity than HuNB1-IgG4. More primate studies are needed to determine the appropriate dosage for the bispecific antibody to be used in future clinical trials [87].

### 3.5 HMBD004 (CD47xCD33)

HMBD004 is an anti-CD47/CD33 bsAb. It is constructed with a highly specific anti-CD47 variable domain arm and the anti-CD33 gemtuzumab variable domain arm [88]. CD33 can be found on all myeloid cells but is significantly over-expressed in AML patients, and its expression positively correlates with stage of the disease [89], [90], [91].

In vitro, HMBD004 resulted in increased phagocytosis of AML cells and prevented significant hemagglutination of erythrocytes. HMBD004 was found to preferentially bind to CD47 + CD33 + cells in a mixture of CD47 + cells. Treatment of an AML xenograft model showed significant decrease in tumor burden and prolonged survival. There are currently no clinical trials testing dosage or effect of HMDB004 in humans [88].

### 3.6 IBI322 (CD47xPD-L1)

IBI322 is an anti-CD47/PD-L1 bsAb which aims to harness both innate and adaptive immune responses by targeting two immunoinhibitory checkpoints [92]. IBI322 consists of a Fab anti-CD47 arm, and a 2-VHH anti-PD-L1 arm. A high affinity to PD-L1 and lower affinity to CD47 allowed IBI322 to selectively bind CD47 + PD-L1 + double positive tumor cells, even in the presence of CD47 + RBCs. IBI322 induced efficient macrophage phagocytosis compared to isotype and monovalent anti-CD47, in the presence of an excessive RBC population. In a Raji-PDL1 lymphoma mice model with human PBMCs, potent tumor inhibition was observed in IBI322 treated group. Additionally, toxicity profile was tested in cynomolgus monkeys. Compared to Magrolimab, lower toxicity was seen in IBI322 treated group represented by much milder adverse effects in RBC and hemoglobin levels [92]. Thus, the imbalanced affinity design of the IBI322 bsAb demonstrated selectivity, efficacy, and minimal toxicities. Currently, multiple clinical trials are underway to study the safety, tolerability, and efficacy of IBI-322 in cancer patients with solid and hematologic malignancy. Among these, a Phase I clinical trial which will investigate IBI322 as a monotherapy in patients with hematologic malignancies who failed standard treatment (NCT04795128).

## 4 Perspectives and conclusions

Development of CD47-targeted agents has become a popular area of pursuit. Blocking the “don't-eat-me” signal overexpressed by tumor cells increases phagocytosis and killing by macrophages. However, growing studies are recognizing the toxicities associated to targeting CD47, whose ubiquitous expression causes off-target killing of non-cancerous cells, especially RBCs and platelets resulting in hemagglutination and anemia. Moreover, the wide expression of CD47 creates an “antigen sink” preventing the treatment from reaching target cells in the desired quantity and decreasing the on-target efficacy. For example, a phase 1 study, of CC‐90002 as monotherapy in AML and MDS (NCT02641002), was terminated due to an insufficient profile for further dose escalation. Thus, there is an ongoing need to exploit safer solutions to overcome toxicities.

Increasing number of novel formats are being investigated to overcome the challenges of life-threatening hemotoxicity. Some approaches discussed in this review include (1) selecting for clones with lower RBC binding and crosslinking, (2) including a bi-specific design targeting a tumor-associated antigen while decreasing CD47 affinity, (3) forgoing the functional Fc portion to reduce uncontrolled engagement and activation of macrophages [93].

A major route for clinical translation for CD47-targeted strategies focus on combination therapy, especially in relapsed/refractory diseases. An array of trials are studying the effect alongside front-line therapies, including (1) chemotherapies, (2) immunomodulatory and checkpoint inhibition agents, (3) tumor antigen targeted antibodies such as anti-CD20 for NHL and anti-CD38 for MM, and (4) drugs that increase CD47 expression in tumor cells. In Table 1, we summarize the agents we discuss in this review, as well as the clinical investigations currently ongoing.

**Table 1:** List of clinical trials involving anti-CD47 therapeutic agents in hematological malignancies.

**Drug**	**Antibody type**	**Clinical trial ID**	**Phase**	**Malignancy type**	**Therapeutic strategy**
**Magrolimab** (Gilead)	Humanized anti-CD47 mAb	NCT02678338	Phase I	R/R AML	Monotherapy
		NCT03248479	Phase Ib	R/R AML/MDS	Monotherapy; +Azacitidine
		NCT04313881	Phase III	Untreated high-risk MDS	+Azacitidine
		NCT04435691	Phase Ib/II	Untreated and R/R AML	+Azacitidine/Venetoclax
		NCT04778410	Phase II	Untreated and R/R AML	+Azacitidine/Venetoclax; + MEC; + CC-486
		NCT03922477	Phase I	R/R AML	+Atezolizumab
		NCT02953509	Phase Ib/II	R/R DLBCL	+Rituximab
		NCT03527147	Phase I	R/R DLBCL	+Rituximab/Acalabrutinib
		NCT04541017	Phase Ib/II	R/R T-cell Lymphoma	+Mogamulizumab
**CC-90002** (Celgene)	Humanized anti-CD47 mAb	NCT02367196	Phase I	R/R NHL	Monotherapy; +Rituximab
**AO-176** (Arch Oncology)	Humanized anti-CD47 mAb	NCT04445701	Phase I/II	R/R MM	Monotherapy; +Bortezomib/Dexamethasone
**Letaplimab** (Innovent Biologics)	Humanized anti-CD47 mAb	NCT03763149	Phase I	Advanced solid tumors and Lymphomas	Monotherapy
		NCT03717103	Phase I	Advanced solid tumors and Lymphomas	Monotherapy; +Rituximab
		NCT04485052	Phase Ib	AML	+Azacitidine
		NCT04485065	Phase Ib	Newly diagnosed higher risk MDS	+Azacitidine
**SRF231** (Surface Oncology)	Fully human anti-CD47 mAb	NCT03512340	Phase Ib	Advanced solid and hematologic cancers	Monotherapy
**IMC-002** (ImmuneOncia Therapeutics)	Fully human anti-CD47 mAb	NCT04306224	Phase I	Advanced solid tumors and R/R Lymphomas	Monotherapy
**TG-1801** (TG Therapeutics)	Anti-CD47xCD19 bsAb	NCT03804996	Phase I	B-cell Lymphoma	Monotherapy; +Ublituximab
		NCT04806035	Phase Ib	B-cell Lymphoma and CLL	Monotherapy; +Ublituximab
**IMM0306** (ImmuneOnco)	Anti-CD47xCD20 bsAb	NCT04746131	Phase I	R/R CD20-positive B-NHL	Monotherapy
**IBI-322** (Innovent Biologics)	Anti-CD47/PD-L1 bsAb	NCT04795128	Phase I	Hematologic malignancies	Monotherapy

**Abbreviations:** mAb: monoclonal antibody; bsAb: bi-specific antibody; R/R: relapsed/refractory; AML: Acute myeloid leukemia; CLL: Chronic lymphocytic leukemia; DLBCL: Diffused large B-cell lymphoma; MDS: Myelodysplastic syndrome; MM: Multiple myeloma; NHL: Non-Hodgkin lymphoma;.

## 5 Funding

This research was supported by an award from the National Institutes of Health (NIH) and the National Cancer Institute of the NIH (U54CA199092), as well as the Paula C. and Rodger O. Riney Blood Cancer Research Initiative Fund. Jennifer Sun was supported by the Spencer T. and Ann W. Olin Fellowship for Women in Graduate Study at the Washington University in St. Louis.

## CRediT authorship contribution statement

**Jennifer Sun:** Conceptualization, Writing – review & editing, Visualization. **Yixuan Chen:** Writing – original draft, Writing – review & editing, Visualization. **Berit Lubben:** Writing – original draft, Writing – review & editing. **Ola Adebayo:** Writing – original draft, Writing – review & editing. **Barbara Muz:** Writing – review & editing. **Abdel Kareem Azab:** Conceptualization, Writing – review & editing, Supervision.

## Declaration of Competing Interest

All authors state no conflict of interest.
